# Harvesting Maturity Assessment of Newly Developed Citrus Hybrids (*Citrus maxima* Merr. × *Citrus sinensis* (L.) Osbeck) for Optimum Juice Quality

**DOI:** 10.3390/plants12233978

**Published:** 2023-11-26

**Authors:** Narendra Singh, Radha Mohan Sharma, Anil Kumar Dubey, Om Prakash Awasthi, Ron Porat, Supradip Saha, Chellapilla Bharadwaj, Amitha Mithra Sevanthi, Amrender Kumar, Nimisha Sharma, Nir Carmi

**Affiliations:** 1 Division of Fruits and Horticultural Technology, ICAR-Indian Agricultural Research Institute, New Delhi 110012, India; narendrahorti94@gmail.com (N.S.); akd67@rediffmail.com (A.K.D.); awasthiciah@yahoo.com (O.P.A.); nims17sharma@gmail.com (N.S.); 2 Department of Postharvest Science, ARO, The Volcani Institute, P.O. Box 15159, Rishon LeZion 7505101, Israel; rporat@volcani.agri.gov.il; 3 Division of Agricultural Chemicals, ICAR-Indian Agricultural Research Institute, New Delhi 110012, India; s_supradip@yahoo.com; 4 Division of Genetics, ICAR-Indian Agricultural Research Institute, New Delhi 110012, India; bharadwaj_gen@iari.res.in; 5 ICAR-National Institute of Plant Biotechnology, New Delhi 110012, India; amithamithra.nrcpb@gmail.com; 6 Agricultural Knowledge Management Unit, ICAR-Indian Agricultural Research Institute, New Delhi 110012, India; amrender.kumar@icar.gov.in; 7 Department of Fruit Tree Sciences, ARO, The Volcani Institute, P.O. Box 15159, Rishon LeZion 7505101, Israel; vhcarmi@volcani.agri.gov.il

**Keywords:** BrimA, fruits, postharvest, juice, new hybrids, physico-chemical

## Abstract

The assessment of the optimum harvesting stage is a prerequisite to evaluating the performance of new citrus genotypes. The intrinsic and extrinsic fruit quality traits of citrus fruits change throughout their developmental process; therefore, to ensure the highest quality, the fruit must be harvested at an appropriate stage of maturity. The biochemical changes in terms of total soluble solids (TSS), titratable acidity (TA), TSS/TA ratio, BrimA (Brix minus acidity), and ascorbic acid, in addition to the organoleptic acceptability of 16 new interspecific citrus hybrids, were evaluated in New Delhi (India) during the H1-H8 harvesting stage at 15-day intervals to standardize the optimum harvesting stage. The TA and ascorbic acid content were at a maximum level during the early harvesting stage and declined with time, reaching the minimum level in the last harvesting stage. The TSS, TSS/TA ratio, and BrimA values were found to have an increasing trend up to the last stage in most of the hybrids. The juice content shows an inclining trend during the initial harvesting observations, followed by stable juice content and then a decline. The BrimA was found to be a better predictor for consumer acceptability compared to the traditional maturity index TSS/TA ratio and, thus, harvesting maturity. Specific TSS, TA, and BrimA values, in addition to the juice percentage and ascorbic acid content, corresponding to the highest hedonic score, were judged as the optimum harvesting stage indicators for an individual hybrid genotype. Among the interspecific hybrids, SCSH-9-10/12, SCSH-11-15/12, and SCSH-17-19/13 were found to be superior, having better juice acceptability organoleptic scores (≥6.0) and higher juice content (≥40%). Principal component analysis based on fruit physico-chemical traits could be able to distinguish the optimum maturity stage in all of the citrus genotypes.

## 1. Introduction

Citrus juice is one of the most widely consumed beverages worldwide owing to its unique, refreshing flavor, as well as its nutritional qualities, and the global annual production of citrus fruits was estimated to be 124.24 million tons during the year 2020 [1,2]. It is estimated that industries process more than 40% of the global citrus production while the remainder is sold in the fresh market, which is further subject to household processing (juice extraction) and is usually consumed as delicious fresh juice [3,4]. Citrus fruit juice is known to contribute vitamin C to human nutrition, in addition to supplementing minerals, antioxidants, flavonoids, limonoids, etc. [3,5,6]. The ethnobotanical and pharmacological value of citrus juice has been widely recognized in traditional medicinal systems and ancient literature for treating ailments such as scurvy, constipation, diarrhea, asthma, fever, jaundice, and skin diseases, besides its cultural and socio-economic importance [7,8]. The demand for citrus fruits and processed juice has increased in the recent past due to an improved economic status and a growing health awareness of consumers, along with the advances in production and post-harvest technologies [9]. 

Globally, sweet orange is the leading crop in the citrus group, followed by mandarins [1]. In India, citrus fruits are the third most important fruit crop next to banana and mango, with a total production of 14.07 million tons from an area of 1.06 million ha [10]. Mandarin has the largest share, followed by sweet orange, lime, and lemon, while pummelo and grapefruit are also cultivated to some extent in India. The Indian citriculture still adheres to traditional cultivars such as Kinnow and Nagpur Santra among the mandarins, and Malta, Valencia, Sathgudi, and Mosambi in the sweet orange, while globally, during the last few decades, a paradigm shift has been observed from traditional cultivars to new, improved cultivars which possess improved juice qualities and were developed through target-specific breeding programs during the last few decades. Hybridization has been proven to be one of the most important methods of citrus crop improvement, and several new hybrids are becoming popular worldwide, such as Daisy, Amber Sweet, Minneola, Osceola, Robinson, Nova, Oroblanco, and Melogold, due to their enhanced juice quality [11,12]. However, there is a dearth of improved indigenous high-yielding varieties possessing excellent fruit juice quality and enhanced health-promoting properties with a wider adaptability to Indian climatic conditions, and which contribute to sustainable horticulture. In addition to traditional desirable traits such as a thin peel, high juice content, seedlessness, and enhanced aroma and flavor, the high nutraceutical properties of citrus fruits have also gained the attention of citrus breeders [12,13,14]. Considering the requirements of Indian citriculture, ICAR-Indian Agricultural Research Institute, New Delhi, developed interspecific citrus scion hybrids (*Citrus maxima* Merr. × *Citrus sinensis* (L) Osbeck) during the second decade of 21st century with the aim to improve the nutraceutical properties of citrus fruits, in addition to the processing traits of fruit juice in sweet citrus scion varieties, keeping in view the health issues related with the modern lifestyle. Furthermore, the pummelo is a hardy species and is known to have tolerance to biotic and abiotic stresses. Thus, the hybrid’s fruit trees may be suitable for changing climate scenarios due to having a wider adaptability. 

Citrus fruits are non-climacteric, showing minimal compositional changes following harvest [15,16,17]. Thus, citrus fruits are harvested when they are ready to eat. Any deviation from the proper maturity stage causes a loss of sensory qualities, thus affecting the marketability of the fruits and their juice processing. Harvesting fruits too early may lead to an undeveloped flavor and the lack of a desirable blend of sweetness and sourness. At the same time, late harvesting tends to cause an insipid taste and reduced shelf life, making the fruits prone to physiological disorders during post-harvest handling. Thus, harvesting citrus fruits at the proper stage of maturity is vital to ensure the palatability (taste, flavor, and color), consumer acceptability, and storability of fruits [18,19].

Many physical and chemical changes are undergone in developing fruit which can be used to assess the optimal harvesting stage. Nevertheless, no single parameter is reliable individually for determining the harvest maturity; hence, usually, a combination of physical and chemical parameters is used to determine the optimum harvesting maturity. The minimum maturity determination of citrus fruits is based on their juice content (lemon and lime), their soluble solids content, their titratable acidity, and the ratio of the latter two (orange, grapefruit, and mandarin) [20]. However, commercial maturity indices in citrus fruit vary and depend on the prevailing edaphoclimatic conditions and cultural practices [17,20,21]. The total soluble solids (TSS) to titratable acidity (TA) ratio (TSS/TA ratio) is the most commonly used criterion for assessing citrus fruit maturity [18,20,22]. However, it has been ascertained that the TSS/TA ratio does not correlate well with the taste perception of fruit juice, as the same ratio may be calculated from different TSS and TA levels, leading to different organoleptic scores for the same ratio [23,24]. The Floridian citrus industry developed variable TSS/TA ratios for different TSS ranges [25]. However, additional indices such as the peel’s colors in particular region(s), juice content, and other physico-chemical attributes may also be considered for judging the optimum maturity stage for harvesting [18,22]. Recently, a new maturity index, ‘BrimA’ [=Brix − k (acid)], has been proposed as a better indicator for organoleptic properties and flavor, and for assessing the harvesting maturity of citrus fruit. In the BrimA index, the TSS is subtracted by the TA and multiplied by a constant which is dependent on the fruit type. The TSS/TA becomes excessively high/low compared to the BrimA due to the former being calculated as a ratio rather than in a subtractive calculation, as is BrimA [20,23,24]. This index has been adopted for commercial harvesting determination in citrus-growing areas of New Zealand, Australia, and California. It is known by various names, as the New Zealand Navel Orange Grade Standard, the Australian Citrus Standard (ACS), and the citrus industry index, respectively [26,27,28]. 

Furthermore, the fruit maturity stage also affects the flavor, aroma, vitamins, and nutritional and nutraceutical benefits, as all of their availabilities change rapidly with the maturity stage due to continuous metabolic processes [29]. With the progression of fruit maturity and ripening, organic acids, vitamins, antioxidants, and phenols are reduced [30,31]. Thus, the realization of the nutritional benefits of citrus juice in the human diet has shifted the focus to determining the optimum fruit harvesting standards in reference to enhanced nutritional values and sensorial attributes of unique fruits [24,29,31]. 

There is a prerequisite to standardize the maturity indices of newly developed interspecific citrus hybrids to ascertain their precise harvesting stage for the further evaluation of their fruit quality, processing attributes, and nutritional value. Thus, the present investigation was undertaken to determine the fruit harvesting maturity standard(s) of newly developed citrus scion hybrids. 

## 2. Results

### 2.1. Changes in Physico-Chemical Fruit Quality Traits

**Figure 1:**
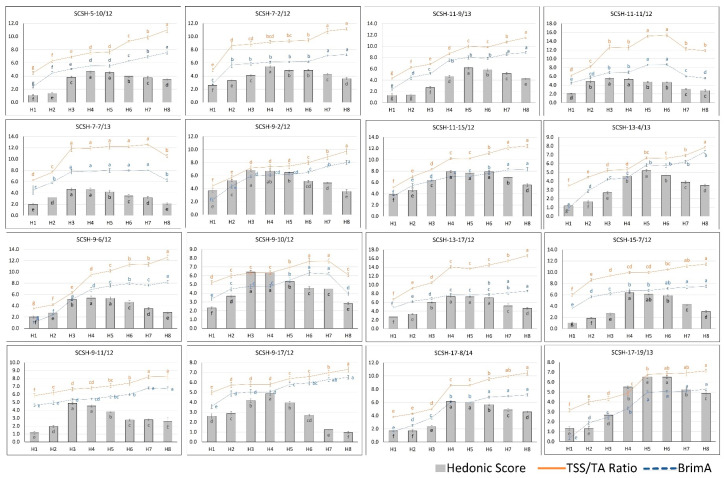
Changes in TSS/TA ratio, BrimA value, and Hedonic score during fruit maturity stage in juice of different interspecific citrus hybrid genotypes. Mean value with different letters (a–g) show a significant difference at *p* ≤ 0.05.

In the present study, changes in the juice content, total soluble solids (TSS), titratable acidity (TA), ascorbic acid, TSS/TA ratio, and BrimA have been observed at various harvesting stages (H1–H8) at 15-day intervals in newly developed interspecific citrus scion hybrids. There was a significant difference in these parameters at different harvesting stages (Table 1 and Table 2; Figure 1). We observed a similar trend among hybrid genotypes for changes in juice (%), i.e., juice content increases during the initial harvesting stages, which reaches a maximum value and then remains quite stable for 2–3 harvesting stages, followed by a decline in the later stages. The juice (%) was found to increase up to the H3 stage in the hybrids SCSH-5-10/12, SCSH-7-2/12, SCSH-9-10/12, SCSH-13-4/13, and SCSH-17-8/14, and after that it remained quite stable for 2–3 harvesting stages followed by declining trends at later harvesting stages. The rest of the hybrids, except hybrid SCSH-11-15/12, reached the maximum level of juice (%) in the H2 stage and remained stable before a decline. The hybrid SCSH-11-15/12 was the last to achieve its highest juiciness, i.e., H5 (1st December), among the evaluated new hybrids (Table 2).

The TSS content of the juice in new scion hybrids varied significantly at different harvesting stages. A total of 13 hybrids (SCSH-5-10/12, SCSH-7-2/12, SCSH-9-2/12, SCSH-7-2/12, SCSH-9-6/12, SCSH-9-11/12, SCSH-11-9/13, SCSH-11-15/12, SCSH-13-4/13, SCSH-13-17/12, SCSH-15-7/12, SCSH-17-8/14, and SCSH-17-19/13), were found to exhibit a continuous increase in their TSS content throughout the harvesting stages from H1 to H8 (Table 1). Although some of the intermediate stages in different genotypes were not significantly different from each other, the overall trend is to be considered as increasing up to the last stage of this experimental study. These hybrids were found to achieve their maximum TSS content in the H8 harvesting stage. In contrast to the above-mentioned hybrid genotypes, three hybrids, namely SCSH-7-7/13, SCSH-9-10/12, and SCSH-11-11/12, showed an increase in their TSS content during the initial stages, and after reaching the maximum, declined sharply. The hybrid SCSH-7-7/13 showed an increase in juice TSS content during the initial stages (H1 and H2) and, after that, remained stable up to H7, followed by a rapid decline, and at the H8 stage, it was found to be statistically similar to the H1. The TSS content in SCSH-9-10/12 increased from H1 to H2 and remained stable up to H4, followed by an increase up to H7, where it peaked, followed by a stiff decline during stages H7–H8 (−2.3°). Similarly, the hybrid SCSH-11-11/12 showed a gradual increase in TSS up to stage H6 and, thereafter, a sharp decline during the later stages of the experimental harvesting (H7 and H8), where its TSS content fell to the extent of 3% and reached the lowest level at H8. All the hybrids showed a very slight difference or gradual changes in their TSS during the H3–H6 stages in the present investigation. In the later stages, most genotypes continued accumulating TSS content and reached their peak in the H8 harvesting stage, except for three hybrids. However, the pace of change and the date of achieving the highest TSS content varied among the hybrids. In some of the hybrids, namely SCSH-7-2/12 SCSH-9-2/12, SCSH-9-6/12, SCSH-9-10/12, SCSH-9-17/12, SCSH-11-9/13 SCSH-13-4/13, SCSH-15-7/12, and SCSH-17-19/13, very high changes (>1%) were observed during the early harvesting (H1–H2) stages, whereas the rest of the hybrids showed a slow rate of increment in their TSS during this period.

In contrast to the TSS content, the titratable acidity (TA) in different citrus hybrids followed a diminishing trend with the approach of maturity (Table 1). The rate of decline was different, as most of the hybrids showed a sharp decline in initial dates followed by very slow changes in the later stages. The hybrids SCSH-9-6/12 and SCSH-17-8/14 registered a significant decline in their TA during the study period from stages H1 to H8, i.e., from 2.04% to 0.86% (−1.18%) and 2.00% to 0.96% (−1.04%), respectively. However, the lowest value of TA was observed on 30th December in all the hybrids.

The TSS/TA ratio, the most commonly used criterion for judging the harvesting maturity of citrus fruits, showed an upward trend in most of the hybrids until the last harvesting stage. However, the changes in the intermediate stages showed differences among the hybrids, as some showed continuous gradual increases. In contrast, others showed a rapid incline followed by a stable TSS/TA ratio during various harvesting stages. However, the highest value for the three hybrids was obtained before, and declined slowly during, the later harvesting stages (Figure 1). The hybrids SCSH-7-7/13 and SCSH-9-10/12 reached the highest TSS/TA ratio at stage H7, followed by a decline, whereas hybrid SCSH-11-11/12 declined the same after H6 harvesting. The rest of the hybrid genotypes showed their highest TSS/TA ratio at stage H8 (Figure 1). 

The BrimA (Brix minus acidity) ratio is a relatively new index adopted to assess the harvesting maturity of citrus fruits. The BrimA value also had significant changes for different harvesting stages (H1–H8), similar to the TSS/TA ratio in the juice of novel citrus scion hybrids (Figure 1). Most of the hybrids had their lowest BrimA at the H1 harvesting stage, reaching the maximum at the H8 harvesting stage, although some of the hybrids exhibited a relatively stable BrimA during the intermediate stages. Similar to the TSS/TA ratio, hybrids SCSH-7-7/13, SCSH-9-10/12, and SCSH-11-11/12 showed a decline in their BrimA after H6 harvestings. However, the trend in the BrimA ratio is similar to that of the TSS/TA ratio in the present investigation (Figure 1), although the changes between various stages were not as drastic/stiff as those seen in the TSS/TA ratio.

The ascorbic acid content followed a diminishing trend in all of the new citrus hybrid genotypes that were studied (Table 2). It showed continuous decline throughout the stages; the lowest was recorded at the last stage of harvesting (H8). Furthermore, at the H1 stage, the ascorbic acid content in the hybrids ranged from 44.95 to 76.28 mg/100 mL, and most of the hybrids had ≥50.0 mg/100 mL ascorbic acid content at this stage. At the last harvest stage (H8), it varied from 23.22–58.87 mg/100 mL in the juice of new citrus hybrids, and most of the hybrids had an ascorbic acid ≤ 50.0 mg/100 mL at this stage.

### 2.2. Consumer Preference Evaluation and Optimum Maturity Stage

The consumer preference for juice at different harvesting stages of citrus hybrids was evaluated by organoleptic sensory evaluation using a hedonic score 1–9 scale with 15 panelists. The hedonic score for the genotypes increased first and then remained stable for about 15–30 days, and afterward, it declined (Figure 1). 

The hybrids SCSH-5-10/12, SCSH-9-6/12, SCSH-13-17/12, SCSH-15-7/12, and SCSH-17-8/14 had their highest hedonic scores during the H4 and H5 harvesting stages; the hybrids SCSH-7-7/13, SCSH-9-2/12, SCSH-9-10/12, SCSH-9-11/12, and SCSH-11-11/12 had their highest acceptability scores from the H3 and H4 harvesting stages; SCSH-17-19/13 showed its highest hedonic score from the H5 and H6 harvesting stages. The hybrids SCSH-7-2/12 and SCSH-9-17/12 had their highest acceptability during the H4 harvesting stage, while the SCSH-11-9/13 and SCSH-13-4/13 citrus hybrids showed their optimum organoleptic acceptability at the H5 harvesting stage. Moreover, SCSH-11-15/12 was the only hybrid which showed a significantly higher hedonic score for juice acceptability during the three consecutive harvesting stages from H4 to H6. 

**Figure 2:**
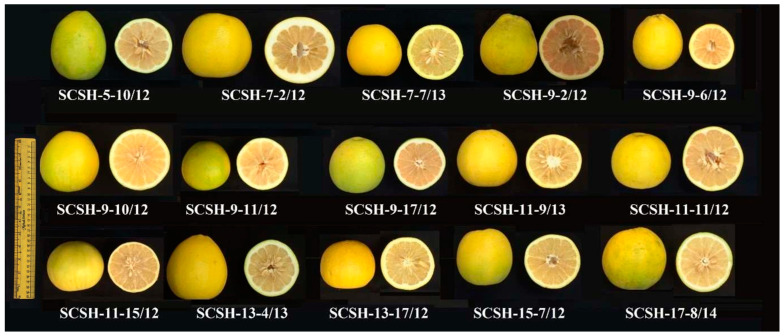
Fruits at optimum harvesting maturity of new interspecific citrus scion hybrids (*Citrus maxima* Merr. × *Citrus sinensis* (L.) Osbeck) evaluated under present study.

The harvesting stage(s) for which individual genotypes achieved the highest hedonic score can be considered the optimum harvesting stage. The fruit attributes at this stage are useful as indices for judging the optimum harvesting, shown in Table 1 and Table 2; Figure 1 and Figure 2.

The hybrid SCSH-17-19/13 was found to have a highest hedonic score of 6.53 (at H5), with 6.78 and 4.98 BrimA, while hybrid SCSH-17-8/14 achieved a highest hedonic score of 8.59 (at H4), which coincides with a TSS/TA ratio of 6.03 and BrimA value of 6.17. The comparison of these hybrids shows that SCSH-17-8/14 had a higher hedonic score compared to SCSH-17-19/13 but had a lower TSS/TA ratio, while the BrimA ratio was found higher hedonic score. Similarly, this trend can also be seen when comparing the hybrids SCSH-11-15/12 and SCSH-11-11/12. This indicates the superiority of BrimA over the TSS/TA ratio.

### 2.3. Relations of Fruit Physico-Chemical Parameters and Hedonic Score

**Figure 3:**
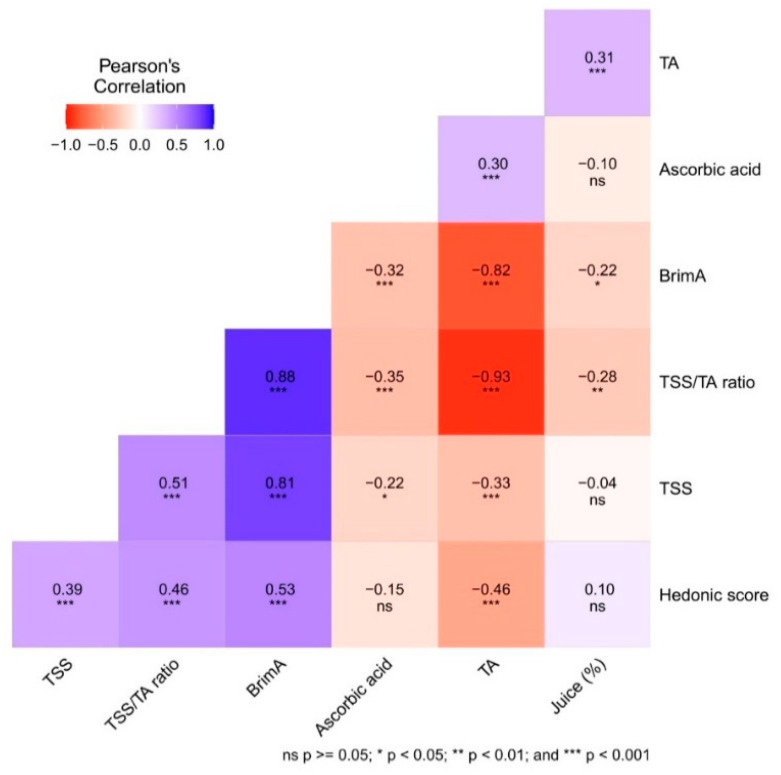
Pearson’s correlation for the fruit physico-chemical traits and hedonic score. TSS= total soluble solids; TA = Titratable acidity; BrimA = Brix − (3 × TA).

Pearson’s correlation was used to study the relationship among various physico-chemical parameters and hedonic scores at different fruit maturity stages among the tested citrus hybrids (Figure 3). The TSS, TSS/TA ratio, and BrimA were positively correlated, while these attributes were negatively correlated with the TA and ascorbic acid content. Further, the TA had a significant positive correlation with ascorbic acid and juice content. Interestingly, the hedonic score did not correlate significantly with the ascorbic acid content and juice (%). However, the organoleptic acceptability judged as a hedonic score was positively associated with the TSS, TSS/TA ratio, and BrimA, and negatively correlated with the TA. Notably, the correlation analysis indicated that the BrmA had the highest correlation (0.53) with the hedonic score compared to the TSS/TA ratio (0.46) and other parameters. 

The coefficient of determination (R^2^) was calculated from the linear regression of the hedonic score with the TSS/TA ratio and BrimA for different harvesting stages in each of the citrus hybrid genotypes (Table 3). The value of R2 was found to be higher for the hedonic score with the BrimA than the hedonic score with the TSS/TA ratio for all of the investigated genotypes.

### 2.4. Discrimination of Harvest Maturity Stages Based on Principal Component Analysis (PCA) 

**Figure 4:**
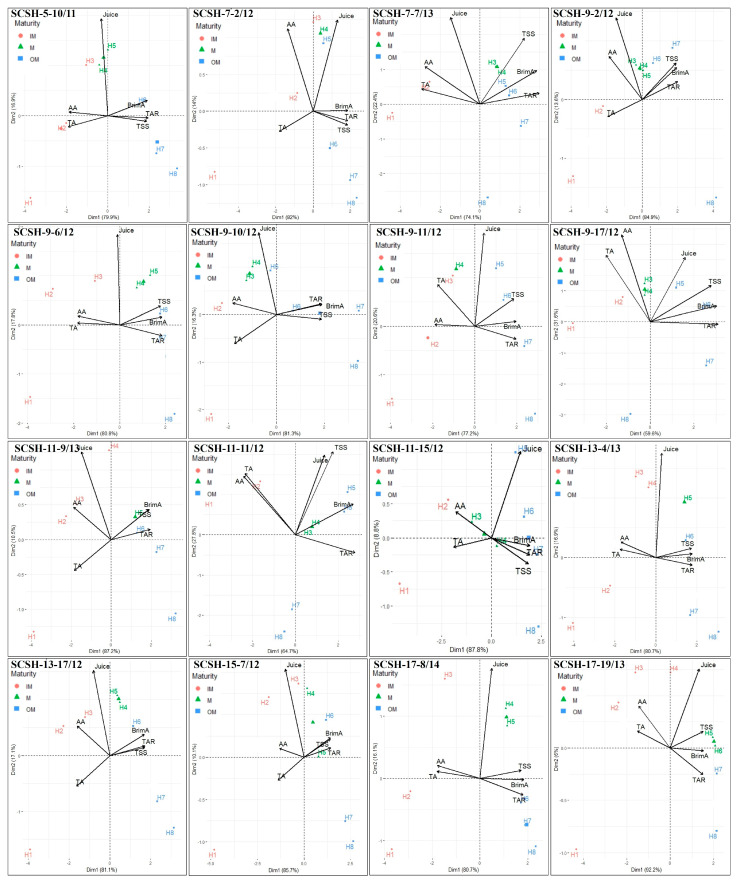
PCA biplots of the maturity-dependent variables in the interspecific citrus scion hybrids. TSS = total soluble solids; TA = titratable acidity; TAR = total soluble solids to titratable acidity ratio; BrimA = Brix-acid; AA = ascorbic acid; Juice = juice (%) (*w*/*w*). IM = premature; M = optimum mature; OM = post mature. Three-stage pre-classification of different harvesting stages carried out based on significant highest hedonic score.

Principle component analysis (PCA) has been widely used with fruit quality traits to emphasize variation and bring out strong patterns in data sets. PCA was appliedin this study using the juice content, TSS, TA, TSS/TA ratio, BrimA, and ascorbic acid content data obtained for different harvesting stages in each citrus genotype (Figure 4). Furthermore, the eight harvesting stages (H1–H8) have been classified into three groups based on significant hedonic score levels, as depicted in Figure 4. These stages are named premature (PM) (harvesting stages prior to highest hedonic score), optimum mature (OM) (harvesting stage/stages corresponding to the highest hedonic score), and postmature ‘PM’ (stages post optimum maturity). The PCA result showed more or less similar PCAs for all of the studied citrus hybrids (Figure 4), and all the genotypes showed only two significant PCs (principal components) which had an eigenvalue ≥1; these components explain ≥90% of the variation. The PCA biplot between PC1 and PC2 could clearly distinguish the hedonic score group of the maturity stage, as these stage(s) were clustered together in the biplot in all of the studied genotypes (Figure 4). The PCAs for all of the genotypes had almost similar results regarding the importance of the different parameters for different dimensions. The TSS, TSS/TA ratio, and BrimA had the highest positive loading value, and the TA and AA showed high negative values for PC1, whereas the juice (%) trait was found to be the most important for PC1, as evidenced by a high loading value (Figure 4). 

Furthermore, the PCA results also showed interrelation among physico-chemical traits. They showed a positive association between the TSS, TSS/TA ratio, and BrimA ratio; these traits are negatively associated with the ascorbic acid content and TA. The results of the PCA for each hybrid genotype showed that multivariate analysis using the above-mentioned traits could differentiate the optimum harvesting stages among the different harvesting stages.

## 3. Discussion

### 3.1. Harvesting Fruit Maturity

The present study concluded that the total soluble solids (TSS) content in all of the citrus hybrid genotypes increased during the later stage of fruit development up to maturity. However, it declined slightly in a few genotypes in the last stages. Contrary to the TSS, the TA showed a downward trend during the later stages of fruit development in all of the hybrid genotypes under study. These findings are in agreement with previous experimental findings on different citrus fruits [31,32,33,34]. 

Towards the maturity stage, the TSS in fruit juice tends to increase due to sugar accumulation [17,35]. Citrus fruits do not have starch. Thus, their sweetness does not increase after harvesting, underpinning the importance of identifying the appropriate levels of sugars at harvesting [16]. The different hybrid genotypes have different rates of increase in their TSS during fruit development due to genetic differences in the rate of sugar accumulation, as enzymes control metabolic activities [36,37]. Additionally, the TSS content serves as a simple, practical guide for harvest in rural areas where growers do not have any other means to measure the maturity index [16]. The worldwide standards set for different oranges determined a minimum of 9% TSS and a minimum TSS:acidity ratio of 8, while grapefruit should have a minimum TSS content of 8%. Notably, all of the hybrids in the present study possess a TSS ≥ 8.0% at fruit maturity [20]. 

The TA is responsible for sourness in fruit juice; it should be lower at fruit maturity, but a lack of acid renders fruit flat and insipid in taste [16,29,36,38]. The organic acids increase during the early fruit growth stage, and diminish towards attaining fruit maturity in citrus fruit [16]. The decline in organic acids at fruit maturity is attributed to the dilution effect and/or their utilization as respiration substrates in different metabolic processes [16,33,36]. Furthermore, the utilization of organic acids, predominantly citric and malic acids, through the TCA (tricarboxylic acid) cycle and several other interconnected metabolic processes contributes to the flavor and synthesis of aromatic compounds at fruit maturity [17,32,36,38]. 

Similar to the TSS/TA ratio, the BrimA also showed an increasing trend during the harvesting stages that were studied. The changes in the TSS/TA ratio evident from the present study are due to the accumulation of sugars in juice sacs and the simultaneous decline in the organic acids, resulting in an increase in the TSS/TA at fruit maturity [32,34,35]. The TSS/TA ratio is the most common determinant for the optimum stage of fruit maturity of citrus fruits and their palatability [16,18,37]. 

Exceptionally, two hybrids showed a decline in their TSS/TA ratio and BrimA during the last stages, which corresponded to a higher loss in their hedonic score during these stages compared to other stages, and both the hybrids exhibited early maturity i.e., reached the highest hedonic score early and if continued to allow to grow on the tree would exhibit a depletion of their sugar reserve at over-maturity [31]. 

The consumer acceptability of the juice of citrus hybrids at different harvesting stages was assessed as a hedonic score on a 1–9 scale. The hedonic score indicates the overall consumer acceptance of fruit juice based on all of its attributes, such as taste, aroma, flavor, and texture. The results of our study indicated that the hedonic score is increased significantly during the initial harvesting stages until maturity, followed by being stable for one or two harvesting stages, and then declining in later harvesting stages. These results are in accordance with previous findings on citrus fruit [24,29,31,34]. The highest TSS/TA ratio, found at the H8 stage in the present investigation, does not coincide with the maximum consumer acceptability; thus, there is a need to identify optimum maturity indices for each genotype/cultivar which provides the best consumer acceptability and flavor. It is to be noted that the initial phase of increase in the hedonic score coincides with an increasing TSS, TSS/TA ratio, and BrimA and a decreasing TA and ascorbic acid content. Contrastingly, after achieving the highest hedonic score, the score starts to decline for all of the hybrid genotypes. However, during this phase, the TSS, TSS/TA, and BrimA continue to rise, and the TA and ascorbic acid content decreases.

Furthermore, the diminishing in the organoleptic score coincides with the acidity reducing to below a level that varies from different hybrids, and individual genotypes have different behaviors, which signifies the role of balance between sweet and acidic taste, as increased sweetness does not follow the trend of acceptability and consumer preference. In addition to lower acidity during late harvesting, Bai et al. [29] also reported, in oranges, that important aroma compounds occurred at the highest concentrations in the middle-to-late season but occurred at lower concentrations at the end of the season; thus, despite an increasing TSS, TSS/TA ratio, and sweetness, the reducing hedonic score is justified.

Among the studied genotypes, SCSH-9-2/12, SCSH-9-10/12, SCSH-11-9/13, SCSH-11-15/12, SCSH-13-17/12, SCSH-15-7/12, SCSH-17-8/14, and SCSH-17-19/13 achieved hedonic scores ≥6.0. Thus, these hybrids showed promise for their taste and consumer acceptability. 

The stage(s) corresponding to the highest organoleptic score for individual hybrids may be considered the optimum maturity stage, as suggested by previous studies [18,24]. The hybrids evaluated in the present study showed optimum harvesting maturity from the H3 to H5 harvesting stages, corresponding to the dates from October 15th to November 15th under the Delhi conditions. Several experimental studies also reported more or less similar maturity periods of pummelo, sweet oranges (parental genotypes), and grapefruit in the Northern Hemisphere [16,22]. Among the studied citrus hybrid genotypes included in the present study, six hybrids (SCSH-7-7/13, SCSH-9-2/12, SCSH-9-6/12, SCSH-9-10/12, SCSH-9-11/12, and SCSH-11-11/12) had the earliest harvesting maturity; seven hybrids (SCSH-5-10/12, SCSH-7-2/12, SCSH-9-17/12, SCSH-11-15/12, SCSH-13-17/12, SCSH-15-7/12, SCSH-17-8/14, and SCSH-17-19/13) reached their optimum harvesting at mid-season. In contrast, the hybrids SCSH-11-9/13 and SCSH-13-4/13 reached their optimum harvesting state late in the present study. Although the hybrids belong to the same parentage pedigree, they are quite different in their behaviors at maturity stages and have different maturity stages. The fruit quality attributes of most of the new interspecific hybrid genotypes are intermediate to the parental genotypes, while a few hybrids showed superior traits, similar to the maternal parents, sweet orange cv Mosambi. However, the TAs of hybrids were in a higher range compared to the acidity of pummelo and Mosambi at harvesting maturity. Each hybrid has different maturity standards and is unique in this sense. It was also reported previously that the progeny of the same parentage have different fruit quality attributes and thus have different harvesting maturity standards [39,40,41,42,43]. This is attributed to the high heterozygosity of parental genotypes (pummelo and sweet orange) in perennial citrus fruits, which leads to transgressive segregation of genes in the progeny and wide variability in the behaviors of the progeny [32,33,44]. The individual hybrids have unique fruit attributes at maturity, resulting from their genetic makeup and leading to differential rates of cell enlargement, albedo tissue development, and juice vesicle development [37].

The hedonic scores of the hybrids showed that, in all of the hybrids, the just-next-to-optimum maturity stage had a higher juice acceptability than the stage preceding the optimum maturity. Thus, this signifies that it is preferable for citrus fruit to be harvested a stage later compared to harvesting early. Thus, fruit harvested at optimum maturity had the highest acceptability, followed by the later stage compared to the before-optimum-maturity stages. As citrus fruits are non-climacteric fruit, they do not show improvement in fruit quality after harvesting [20,31].

The hedonic score remains quite stable for 15–30 days in the new hybrid citrus fruits, which agrees previous findings on tree storage, and which is unique in citrus fruits although they are non-climacteric. The fruit on the tree remains longer, which helps sustain their quality compared to fruit harvested and kept in cold storage. Among the studied hybrid genotypes, only four hybrids, namely SCSH-7-2/12, SCSH-9-17/12, SCSH-11-9/13, and SCSH-13-4/13, showed optimum maturity for only one harvesting stage; thus, these hybrids are not suitable for long-keeping quality with on tree storage. The rest of the hybrids can be harvested in the window period of one month or more, except hybrid SCSH-11-15/12, which, exceptionally, retained its highest acceptability during three harvesting stages (H4, H5, and H6), which is an advantageous attribute of this unique hybrid. This attribute of hybrid fruit will help growers and processors as it provides a longer window of harvesting to the growers, fetches better market price options, and saves energy for low-temperature storage.

However, the physical attributes of citrus fruit are not a reliable criterion for maturity as they change in response to competition among the fruits, rootstock, nutritional status, water balance, and prevailing edaphoclimatic conditions [16]. Juice content is an important criterion for selecting a new suitable hybrid for economic juice processing, and it is also considered as useful as a maturity index in citrus in addition to the TSS/TA ratio to judge optimum maturity [16,18,20,24]. Hence, our findings regarding fruit quality attributes help in supplementing maturity criteria. 

The juice content in the hybrid genotypes during different harvesting stages showed varied patterns. In general, it was found to increase slowly during the earlier harvesting stages; after reaching the maximum, it started to decline gradually. Similar findings were reported in citrus fruits previously by Manzoor et al. [31] and Muhtaseb [45]. The results for the juice content also emphasize the importance of the optimum harvesting stage, as any deviation from this leads to a loss in the juice content, which is the major economic consideration for juice processing industries. In the present study, the juice content in different citrus hybrids at the maturity stage varied from 22.91% to 48.24%. A total of 10 hybrids had juice content ≥ 33%, the prescribed minimum standard for the grapefruit and sweet orange cultivars in the USA and several other countries [16]. Further, the juice content might decrease with delayed harvesting; thus, fruit should be harvested at the optimum maturity [16,24,27]. 

Significant variation was also recorded amongst the interspecific citrus hybrid genotypes in the ascorbic acid contents in the fruit juice. Citrus fruits are known for their high ascorbic acid content, antioxidant activities, and different bioactive compounds. The changes in ascorbic acid content at different harvesting stages in the present study and its range at the optimum maturity stage is comparable with earlier findings [34,46]. The amount of ascorbic acid in the fruit increases at first and then drops as the fruit ripens, which further emphasizes the need for the optimum maturity stage to balance a good flavor and sugar-acid blend with ascorbic acid content [16,36]. The ascorbic acid content in sweet orange juice increases up to 95 days after anthesis, followed by a continuous diminishing trend during fruit ripening, which is due to involvement in metabolic pathways and a dilution effect [46,47]. 

In citrus fruits, during maturation and development, the peel color changes from green to yellow, orange, or orange-red as per the genetic character of the variety under favorable climatic and growing conditions [17,48]. All the interspecific citrus scion hybrids had yellow peel at the optimum fruit maturity stage (corresponding to the highest hedonic score). However, fruit color is not a reliable index for determining citrus fruit maturity, as prevalent climatic conditions have a significant role in peel color development [11,18,37,48]. In countries like India, the peel color is an important consideration during fruit grading and marketing. As per standards in the USA, grapefruit (a hybrid of pummelo × sweet orange) should have a yellow color on two-thirds of the fruit surface. Further, green should not exceed one-fifth of the total surface for sweet orange superior grades. Notably, the genotype which has the best coloring in the natural environment excludes the degreening process. 

### 3.2. Correlation among the Fruit Physico-Chemical Attributes at Fruit Maturity

The results of the present study indicated a significant correlation between different fruit physico-chemical attributes and hedonic scores. The correlation in the present study shows how the different traits increase or decrease during different harvesting stages. A significant positive correlation of hedonic score with the TSS, TA, TSS/TA ratio, and BrimA indicates these parameters’ importance in assessing the organoleptic sensory properties and judging the optimum quality of fruit. These findings are in agreement with previous studies on the different citrus fruits pummelo, mandarins, and grapefruit [14,20,24]. 

A higher correlation between the hedonic score and BrimA compared to the TSS/TA ratio and the hedonic score indicates that the BrimA is a better predictor of acceptability than the widely used TSS/TA ratio. This result is further confirmed by greater linearity in the relationship of the BrimA with the hedonic score in each citrus hybrid. The results agree with the previous findings of Jordan et al. [23], Obenland et al. [24], and Clark [26]. Although the previous studies carried out by Obenland et al. [24] suggested better suitability of BrimA for late-season harvesting in oranges, our findings also found its suitability in the early season. The BrimA ratio has not been used or standardized for Indian citriculture to date. Hence, this study will pave the way for adding a new maturity standard, BrimA, for Indian citriculture in judging the maturity indices of citrus fruits.

Although the TSS/TA ratio and BrimA ratio showed a similar pattern, the TSS/TA ratio at different stages showed drastic changes of high magnitude, which is due to its method of calculation, as TSS divided by the very small value of TA and thus a slight change in the TA or TSS leads to large-magnitude changes in the TSS/TA ratio. On the other hand, changes between different stages are less remarkable than the TSS/TA ratio, as depicted in Figure 1. This is due to its calculation/formula, as it includes higher weightage to the changes in the TA, as it is multiplied to a constant, i.e., three, in the present case of citrus fruits, and the BrimA is calculated by subtracting rather than dividing. The rationale behind introducing the BrimA as a new maturity index and its superiority for predicting juice acceptability can be explained by previous studies that human sensory faculties are more sensitive to acidity or tartness compared to sweetness, and a slight change in the TA can significantly affect taste compared to changes of the same magnitude in the sweetness [23,24].

### 3.3. Discrimination of Harvest Maturity Stages Based on Principal Component Analysis (PCA) 

PCA based on the juice (%), TSS, TA, TSS/TA ratio, BrimA, and ascorbic acid content attributes clearly distinguished the different maturity stages in citrus in the PC1 and PC2 biplots. PC1 contributed very high variation across the genotypes (≥68%). Moreover, the TSS, TA, TSS/TA ratio, and BrimA were invariably the most important traits for optimum maturity determination, with a high loading score for PC1, followed by jJuice (%), being the highest contributor to PC2. Furthermore, PCA also showed the interrelationship between the fruit traits which play an important role in citrus harvesting maturity. Previously, PCA has been successfully employed for the assessment and differentiation of maturity stages in citrus [31], mango [34], grapes [49], and peach [50]. These studies support the present study’s findings for multivariate analysis for distinguishing the harvesting maturity of citrus fruits. 

## 4. Material and Methods

### 4.1. Study Site 

**Figure 5:**
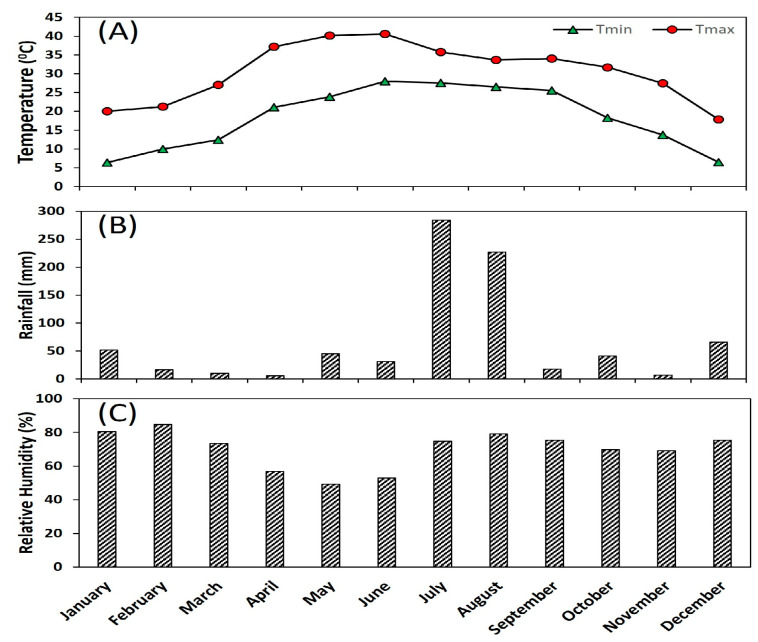
Mean monthly weather parameters at the experimental location. (**A**) Tmax = maximum temperature, Tmin = minimum temperature, (**B**) average rainfall, (**C**) average relative humidity.

The experiment was conducted at the Experimental Farm of ICAR—Indian Agricultural Research Institute, New Delhi (28°64’ N and 77°15’ E; 228 m above the mean sea level). The experimental site is part of the Trans-Gangetic plains, which have a typically subtropical climate characterized by dry and hot summers followed by cold winters. Weather parameters at the experimental location during the studied period are presented in Figure 5.

### 4.2. Plant Materials

In the present investigation, 16 new interspecific citrus scion hybrids (*C. maxima* [Burm. f.] Osbeck × *Citrus sinensis* [L.] Osbeck) were undertaken for the study (Table 4). The hybridization was carried out by using the traditional method of emasculation, followed by controlled pollination between pummelo (*C. maxima* [Burm. f.] Osbeck) as the seed parent and sweet orange cv. Mosambi as the pollen parent. The seeds obtained from crossed fruits were sown in the nursery and later planted in the field as seedlings during 2012–2014 at 5 m × 5 m spacing. The trees were subjected to uniform cultural practices during the investigation. The new hybrids started bearing fruit from the year 2017 onwards. Out of the large hybrid population after initial screening during the year 2018, a total of 16 hybrids were selected for the standardizing maturity indices (Table 4).

Each interspecific hybrid is a unique genotype and was maintained as a single plant. A total of 12 fruits, three from each direction (north, south, east, and west), were hand-picked randomly from each hybrid tree to determine optimum maturity at regular intervals of 15 ± 1 days starting from 15th September up to 30th December 2019. These harvesting stages were denoted as H1–H8, respectively. Fruits were transferred to the analysis lab, and all the analyses were performed on the same day on fresh fruit samples. The fruit was wiped and cleaned with a moist cloth, then air-dried at room temperature until excessive moisture evaporated from the skin prior to measurement. The average value obtained from 12 fruit samples (3 each from different directions of the tree) for the data on fruit traits in conjunction with organoleptic evaluation was used for judging the optimum harvesting stage for new interspecific citrus hybrids fruits.

### 4.3. Estimation of Juice Content

The three fruits from different directions (west, north, east, and south) of a hybrid tree were considered a single replication, and analyses were performed in quadruplicate. Fruit weight was recorded using a digital weighing balance (CTG 3101 Precision Balance, Citizen Scale Pvt. Ltd., Mumbai, India; Sensitivity 0.1 g). Each fruit was cut in the equatorial area, and juice was squeezed from these fruits with the help of a hand juice extractor. The extracted juice from each fruit was taken separately, strained through a sieve (2 mm), and weight was noted. The juice percent (*w*/*w*) was calculated as the following formula:

(1) $$Juice\left(\%\right)=\left(\frac{Juice\;weight\;in\;g}{Fruit\;weight\;in\;g}\right)\times 100$$

### 4.4. Biochemical Attributes

The biochemical analyses (TSS, TA, TSS/TA ratio, BrimA, and ascorbic acid) were performed at each harvesting stage on the fruit juice immediately after cutting the fruit in quadruplicate, where an average of three fruits was considered a replication. Total soluble solids (TSS) content was measured with the help of a hand refractometer (BRIX 0–32%, Erma Inc., Tokyo, Japan) at 25 °C. Titratable acidity (TA) was determined by titrating a known volume of fruit juice against 0.1N NaOH and expressed as percent citric acid equivalent [24]. The TSS/TA ratio was calculated by dividing the TSS (%) of the extracted juice by its titratable acidity.

In addition to the above parameters, BrimA (Brix minus Acid) is a relatively new proposed maturity standard and is also accepted for commercial citrus fruit harvesting in different citrus growing areas [20,26,28]. It was calculated as BrimA = Brix − k(TA), where the value of the k constant is taken as three, as Obenland et al. [24] suggested for different fruit stages in all the new hybrid genotypes.

The ascorbic acid content in the juice of new citrus hybrids at different harvesting stages was also determined by the redox titration method using an iodine solution [51]. A total of 20 mL juice was transferred into a 250 mL conical flask, followed by the addition of 150 mL distilled water, 5 mL potassium iodide (0.6 mol L^−1^), 5 mL of HCl (1 mol L^−1^), and 1 mL starch indicator (0.5%). The solution was titrated against the potassium iodate solution (0.002 mol L^−1^). The endpoint of the titration is the first permanent trace of a dark blue-black color. The amount of ascorbic acid (mg/100 mL) in the fresh juice sample was calculated with the titration reaction as 2IO^−3^ + 10I^−^ + 12H^+^ → 6I_2_ + 6H_2_.

### 4.5. Consumer Preference Evaluation and Optimum Maturity Stage

The organoleptic (consumer preference) evaluation was carried out by 15 untrained panelists (ICAR-Indian Agricultural Research Institute, Division of Fruits and Horticultural Technology, New Delhi, India). The fruit obtained from citrus hybrids on different harvesting dates (H1–H8) was cut in two halves, and the juice was extracted with the citrus press juicer. The juice obtained from the quadruplicate fruit sample was strained with the help of a stainless-steel sieve (2 mm) in the coded beaker and three samples for each replication were presented to the panelists in separate booths. The consumer preference response of the panelists has been noted as a 9-point hedonic scale (9 = like extremely; 8 = like very much; 7 = like moderately; 6 = like slightly; 5 = neither like nor dislike; 4 = dislike slightly; 3 = dislike moderately; 2 = dislike very much; 1 = dislike extremely) [52]. The harvest stage(s), coinciding with the highest hedonic score, which is significantly different from the previous and next stage, were considered as optimum maturity stage (M), the previous stages are considered as immature (IM), and later stages are considered as postmature (PM).

The optimum harvesting maturity standards were expressed as the range of fruit physio-chemical traits *viz.*, juice (%), TSS/TA ratio, and BrimA corresponding to the significant highest organoleptic score [18,22,24] and the fruit of these novel hybrids at optimum harvesting stages is depicted in Figure 2.

### 4.6. Statistical Analyses

The experimental data pertaining to the fruit physico-chemical parameters were subjected to analysis of variance (ANOVA) using the statistical software SAS (9.3 SAS Institute, Inc., Cary, NC, USA) and expressed as the mean of quadruplicate (three fruit/replication and total 12 fruits at each harvesting) measurements. The differences in mean values at different harvesting stages in new interspecific citrus hybrids were considered statistically significant at LSD (*p* ≤ 0.05) and expressed in the results as mean ± SEm (standard error of the mean). The relationship amongst citrus fruits’ physical and biochemical parameters was computed using Pearson’s correlation method using RStudio (RStudio, PBC, Version 2022.07.1-554). The coefficient of determination (R^2^), which explains the scattering of the data points around the fitted regression line, was worked out to compare and estimate the association of TSS/TA ratio and BrimA with hedonic score at different harvesting stages in the investigated genotypes [24].

Multivariate analysis as principal component analysis (PCA) was performed as suggested by Kienzle et al. [53] for each hybrid citrus genotype using the fruit physico-chemical traits to distinguish between different harvest maturity stages (H1–H8) using RStudio (RStudio, PBC, Version 2022.07.1-554). The organoleptic score (hedonic score) obtained for different fruit maturity stages was not included in the multivariate analyses to analyze whether the multivariate analysis using studied parameters could differentiate the immature (IM), mature (M), and postmature (PM) stages in all the studied 16 citrus hybrid genotypes.

## 5. Conclusions

The results of this study indicated a gradual increase in the total soluble solids (TSS) content, TSS/TA ratio, and BrimA value during the harvesting stage of fruit development until fruit maturity. In contrast, there was a decline in the acidity and ascorbic acid content with regard to the progressive growth stage of the fruits. The new hybrids have a unique maturity index for optimum harvesting as determined from the highest organoleptic acceptability score and fruit parameters at particular stages. Principal component analysis based on fruit physico-chemical characteristics at different harvesting stages could distinguish the stage corresponding to the highest sensory quality. Further, the BrimA was found to be the best predictor for consumer acceptability and sensory properties at different harvesting stages in all of the genotypes. Among the newly developed citrus hybrids, SCSH-9-10/12, SCSH-11-15/12, and SCSH-17-19/13 were found to be superior in their physico-chemical fruit quality attributes and sensory preference. The determination of maturity standards for new citrus hybrids fulfills the pre-requisite for the further evaluation of their nutraceutical properties, yield potential, optimization of management practices, shelf life, and amenability for processing.

## Figures and Tables

**Table 1:** Changes in total soluble solids (TSS) and titratable acidity (TA) during fruit harvesting stage in juice of different interspecific citrus hybrid genotypes.

**Hybrid Genotypes**	**Total Soluble Solids (TSS) (%)**							
	**H1**	**H2**	**H3**	**H4**	**H5**	**H6**	**H7**	**H8**
SCSH-5-10/12	8.20 ± 0.5 e	8.50 ± 0.53 d	9.00 ± 0.42 c	9.20 ± 0.54 c	9.18 ± 0.53 c	9.27 ± 0.49 c	9.91 ± 0.55 b	10.42 ± 0.57 a
SCSH-7-2/12	7.33 ± 0.4 c	8.80 ± 0.31 b	8.90 ± 0.41 b	9.09 ± 0.4 b	9.10 ± 0.32 b	9.13 ± 0.38 b	9.83 ± 0.37 a	9.93 ± 0.46 a
SCSH-7-7/13	8.65 ± 0.42 c	9.70 ± 0.44 b	10.40 ± 0.75 ab	10.46 ± 0.48 a	10.56 ± 0.56 a	10.52 ± 0.42 a	10.56 ± 0.36 a	8.76 ± 0.55 c
SCSH-9-2/12	7.20 ± 0.39 d	9.2 ± 0.43 c	10.13 ± 0.39 b	10.27 ± 0.4 b	10.29 ± 0.47 b	10.38 ± 0.4 b	11.25 ± 0.35 a	11.57 ± 0.49 a
SCSH-9-6/12	7.18 ± 0.39 e	8.4 ± 0.36 d	9.8 ± 0.38 c	10.07 ± 0.46 bc	10.68 ± 0.46 a	10.8 ± 0.37 a	10.33 ± 0.41 b	10.81 ± 0.4 a
SCSH-9-10/12	7.98 ± 0.33 d	9 ± 0.36 c	9.13 ± 0.4 c	9.1 ± 0.34 c	9.7 ± 0.45 b	10.42 ± 0.4 a	10.16 ± 0.39 a	7.8 ± 0.42 d
SCSH-9-11/12	9.50 ± 0.43 d	9.5 ± 0.46 d	9.67 ± 0.32 cd	9.71 ± 0.33 cd	9.9 ± 0.44 bc	10.06 ± 0.4 b	10.72 ± 0.39 a	10.54 ± 0.42 a
SCSH-9-17/12	9.00 ± 0.55 c	10.25 ± 0.67 b	10.4 ± 0.66 b	10.4 ± 0.59 b	10.95 ± 0.65 a	10.92 ± 0.59 a	11.02 ± 0.57 a	11.05 ± 0.56 a
SCSH-11-9/13	7.80 ± 0.67 f	8.8 ± 0.64 e	9.2 ± 0.55 d	11.5 ± 0.57 abc	11.47 ± 0.6 bc	11.33 ± 0.51 c	11.93 ± 0.56 ab	11.99 ± 0.53 a
SCSH-11-11/12	8.7 ± 0.44 c	9 ± 0.46 bc	9.07 ± 0.45 b	9.13 ± 0.44 b	10.72 ± 0.38 a	10.72 ± 0.39 a	8.03 ± 0.39 d	7.48 ± 0.39 e
SCSH-11-15/12	8.67 ± 0.42 d	9.5 ± 0.54 c	9.76 ± 0.42 c	9.93 ± 0.42 bc	9.97 ± 0.46 bc	10.41 ± 0.47 b	10.92 ± 0.48 a	10.96 ± 0.56 a
SCSH-13-4/13	7.21 ± 0.33 e	8.9 ± 0.43 d	9.86 ± 0.44 c	10.03 ± 0.54 c	10.67 ± 0.38 b	10.6 ± 0.45 b	10.93 ± 0.42 b	11.89 ± 0.44 a
SCSH-13-17/12	8.67 ± 0.53 e	9.23 ± 0.46 d	9.6 ± 0.51 cd	9.8 ± 0.49 bc	9.83 ± 0.5 bc	9.83 ± 0.41 bc	10.16 ± 0.52 ab	10.46 ± 0.48 a
SCSH-15-7/12	7.2 ± 0.35 e	8.63 ± 0.41 d	9.18 ± 0.41 c	9.67 ± 0.45 b	9.68 ± 0.41 b	9.97 ± 0.34 ab	10.08 ± 0.33 a	10.16 ± 0.36 a
SCSH-17-8/14	7.6 ± 0.43 d	8.33 ± 0.48 c	9.2 ± 0.45 b	9.27 ± 0.42 b	9.27 ± 0.44 b	9.91 ± 0.48 a	9.9 ± 0.36 a	10.01 ± 0.49 a
SCSH-17-19/13	6.95 ± 0.35 e	7.6 ± 0.36 d	8.04 ± 0.36 c	8.71 ± 0.35 b	8.93 ± 0.39 ab	8.97 ± 0.42 ab	8.97 ± 0.33 ab	9.12 ± 0.3 a
**Hybrid Genotypes**	**Titratable Acidity (TA) (%)**							
	**H1**	**H2**	**H3**	**H4**	**H5**	**H6**	**H7**	**H8**
SCSH-5-10/12	1.60 ± 0.02 a	1.35 ± 0.02 b	1.30 ± 0.01 b	1.22 ± 0.02 c	1.20 ± 0.02 c	1.00 ± 0.02 d	1.00 ± 0.01 d	0.95 ± 0.01 e
SCSH-7-2/12	1.50 ± 0.03 a	1.02 ± 0.02 b	1.01 ± 0.01 bc	0.99 ± 0.01 bcd	0.98 ± 0.01 cd	0.97 ± 0.01 d	0.91 ± 0.02 e	0.89 ± 0.02 e
SCSH-7-7/13	1.38 ± 0.02 a	1.27 ± 0.03 b	0.88 ± 0.01 c	0.88 ± 0.02 c	0.87 ± 0.02 cd	0.86 ± 0.01 cd	0.84 ± 0.01 d	0.83 ± 0.01 d
SCSH-9-2/12	1.63 ± 0.03 a	1.61 ± 0.02 a	1.41 ± 0.01 b	1.40 ± 0.02 b	1.38 ± 0.02 b	1.30 ± 0.01 c	1.27 ± 0.01 c	1.19 ± 0.02 d
SCSH-9-6/12	2.04 ± 0.02 a	2.01 ± 0.03 a	1.56 ± 0.03 b	1.06 ± 0.01 c	1.05 ± 0.01 c	0.96 ± 0.01 d	0.91 ± 0.01 de	0.86 ± 0.01 e
SCSH-9-10/12	1.54 ± 0.03 a	1.5 ± 0.02 ab	1.45 ± 0.03 bc	1.44 ± 0.02 c	1.4 ± 0.02 cd	1.37 ± 0.02 de	1.33 ± 0.02 ef	1.28 ± 0.01 f
SCSH-9-11/12	1.63 ± 0.02 a	1.53 ± 0.02 b	1.45 ± 0.01 c	1.43 ± 0.02 cd	1.4 ± 0.01 de	1.36 ± 0.02 e	1.31 ± 0.07 f	1.28 ± 0.02 f
SCSH-9-17/12	1.81 ± 0.01 a	1.79 ± 0.02 a	1.79 ± 0.01 a	1.79 ± 0.01 a	1.72 ± 0.02 b	1.66 ± 0.03 c	1.58 ± 0.01 d	1.51 ± 0.02 e
SCSH-11-9/13	1.80 ± 0.04 a	1.40 ± 0.03 b	1.35 ± 0.01 c	1.32 ± 0.02 c	1.15 ± 0.02 d	1.15 ± 0.01 d	1.11 ± 0.01 d	1.04 ± 0.01 e
SCSH-11-11/12	1.40 ± 0.01 a	1.10 ± 0.01 b	0.72 ± 0.03 c	0.73 ± 0.02 c	0.71 ± 0.01 c	0.70 ± 0.01 cd	0.66 ± 0.01 de	0.63 ± 0.01 e
SCSH-11-15/12	1.70 ± 0.02 a	1.35 ± 0.02 b	1.18 ± 0.02 c	0.97 ± 0.01 d	0.97 ± 0.01 d	0.93 ± 0.01 de	0.90 ± 0.01 ef	0.88 ± 0.02 f
SCSH-13-4/13	2.10 ± 0.03 a	2.00 ± 0.02 b	1.89 ± 0.01 c	1.89 ± 0.01 c	1.61 ± 0.01 d	1.61 ± 0.02 d	1.58 ± 0.02 de	1.52 ± 0.02 e
SCSH-13-17/12	1.30 ± 0.03 a	1.00 ± 0.01 b	0.92 ± 0.02 c	0.70 ± 0.01 de	0.72 ± 0.02 d	0.68 ± 0.01 e	0.66 ± 0.02 ef	0.63 ± 0.02 f
SCSH-15-7/12	1.20 ± 0.021 a	1.00 ± 0.012 b	0.98 ± 0.01 bc	0.97 ± 0.01 bc	0.97 ± 0.01 bc	0.95 ± 0.01 c	0.91 ± 0.02 d	0.89 ± 0.01 d
SCSH-17-8/14	2.00 ± 0.03 a	1.94 ± 0.02 b	1.84 ± 0.03 c	1.08 ± 0.01 d	1.08 ± 0.01 d	1.04 ± 0.02 de	0.99 ± 0.01 ef	0.96 ± 0.01 f
SCSH-17-19/13	2.20 ± 0.01 a	1.90 ± 0.03 b	1.86 ± 0.02 b	1.79 ± 0.02 c	1.32 ± 0.02 d	1.31 ± 0.02 d	1.30 ± 0.02 d	1.28 ± 0.01 d

H—harvesting stage. Results are means of four determinations ± SEm. Different letters within a row followed by Mean ± SEm show a significant difference at *p* ≤ 0.05.

**Table 2:** Changes in juice per cent and ascorbic acid content during fruit harvesting stage in juice of different interspecific citrus hybrid genotypes.

**Hybrid Genotypes**	**Juice (%)**							
	**H1**	**H2**	**H3**	**H4**	**H5**	**H6**	**H7**	**H8**
SCSH-5-10/12	36.23 ± 0.97 b	36.93 ± 0.58 ab	37.6 ± 0.85 a	37.56 ± 0.67 a	37.75 ± 0.81 a	37.03 ± 1.08 ab	36.45 ± 1.11 b	36.25 ± 0.9 b
SCSH-7-2/12	21.62 ± 0.51 d	23.33 ± 0.56 c	25.5 ± 0.77 a	25.47 ± 0.78 a	25.5 ± 0.74 a	24.65 ± 0.82 b	24.08 ± 0.63 b	24.05 ± 0.87 bc
SCSH-7-7/13	22.68 ± 0.83 ab	22.91 ± 0.87 a	22.9 ± 0.59 a	22.79 ± 0.53 a	21.77 ± 0.94 ab	21.54 ± 1.35 b	20.28 ± 0.72 c	19.98 ± 0.64 c
SCSH-9-2/12	24.96 ± 0.87 a	25.19 ± 0.5 a	25.15 ± 0.65 a	25.03 ± 0.54 a	25 ± 0.58 a	24.97 ± 0.62 a	24.91 ± 0.73 a	23.48 ± 0.72 b
SCSH-9-6/12	34.54 ± 1.03 bc	35.53 ± 0.69 a	35.51 ± 0.77 a	35.51 ± 0.55 a	35.6 ± 0.89 a	35.23 ± 0.51 ab	35.01 ± 0.55 abc	34.28 ± 0.58 c
SCSH-9-10/12	39.14 ± 1.09 e	40.56 ± 0.89 bcd	41.83 ± 1.11 ab	41.56 ± 0.77 abc	42.08 ± 0.94 a	41.04 ± 0.75 abcd	40.32 ± 0.75 cde	39.76 ± 1.01 de
SCSH-9-11/12	35.75 ± 0.88 b	36.83 ± 0.87 a	36.92 ± 0.77 a	37.03 ± 0.6 a	36.91 ± 0.71 a	36.34 ± 0.64 ab	36.31 ± 1.11 ab	35.64 ± 0.66 b
SCSH-9-17/12	32.88 ± 0.78 b	33.43 ± 1.02 ab	34.24 ± 0.9 a	34.33 ± 0.85 a	34.41 ± 0.95 a	34.16 ± 0.92 a	33.75 ± 1.02 ab	33.04 ± 0.7 b
SCSH-11-9/13	38.76 ± 0.69 ab	39.45 ± 0.78 a	39.42 ± 0.73 a	39.45 ± 1.08 a	38.41 ± 1 ab	38.33 ± 1.19 b	38.01 ± 0.85 bc	37.21 ± 0.76 c
SCSH-11-11/12	37.33 ± 0.9 b	38.87 ± 0.93 a	38.85 ± 1.11 a	38.83 ± 1.21 a	38.86 ± 0.99 a	38.2 ± 0.7 ab	37.28 ± 0.8 b	37.12 ± 1.06 b
SCSH-11-15/12	43.78 ± 0.91 d	45.81 ± 0.66 c	45.87 ± 1 bc	45.86 ± 1.09 bc	47.87 ± 1.12 a	47.03 ± 1.25 ab	46.73 ± 0.88 abc	45.66 ± 0.78 c
SCSH-13-4/13	38.75 ± 1.21 c	39.28 ± 0.61 bc	40.82 ± 1.22 a	40.8 ± 0.93 a	40.72 ± 0.66 a	40.23 ± 1.06 ab	39.15 ± 0.91 bc	39.01 ± 1.14 bc
SCSH-13-17/12	26.45 ± 0.81 b	27.6 ± 0.82 a	27.55 ± 0.84 a	27.38 ± 0.92 a	27.53 ± 0.88 a	27.23 ± 0.66 a	26.03 ± 0.87 bc	25.45 ± 0.69 c
SCSH-15-7/12	24.33 ± 0.85 bc	24.81 ± 0.78 ab	24.85 ± 1.08 a	24.45 ± 0.86 bc	23.67 ± 0.81 c	23.91 ± 1.29 bc	22.43 ± 0.7 d	22.07 ± 0.85 d
SCSH-17-8/14	26.78 ± 0.96 c	27.33 ± 1.04 bc	28.49 ± 1.02 a	28.49 ± 0.87 a	28.33 ± 1.02 ab	27.56 ± 0.87 abc	27.36 ± 1.14 bc	27.21 ± 0.76 c
SCSH-17-19/13	44.32 ± 0.91 b	46.57 ± 1.32 a	47.11 ± 1.37 a	47.95 ± 1.13 a	48.24 ± 1.1 a	48.21 ± 1.3 a	48.01 ± 0.79 a	47.23 ± 0.66 a
**Hybrid Genotypes**	**Ascorbic Acid (mg/100 mL)**							
	**H1**	**H2**	**H3**	**H4**	**H5**	**H6**	**H7**	**H8**
SCSH-5-10/12	72.53 ± 1.57 a	72.17 ± 1.98 ab	68.45 ± 1.7 bc	67.97 ± 3.61 c	63.15 ± 1.84 d	59.13 ± 2.79 e	58.67 ± 1.6 e	57.72 ± 3.1 e
SCSH-7-2/12	69.88 ± 3.14 a	69.99 ± 2.74 a	68.18 ± 1.22 a	67.45 ± 0.93 a	66.42 ± 1.34 a	60.28 ± 1.45 b	60.28 ± 2.49 b	58.87 ± 1.08 b
SCSH-7-7/13	53.23 ± 2.08 a	48.76 ± 1.81 b	42.62 ± 1.65 c	42.52 ± 1.83 c	42.58 ± 1.48 c	41.27 ± 2.11 cd	39.45 ± 1.55 de	37.82 ± 2.27 e
SCSH-9-2/12	76.28 ± 1.13 a	73.26 ± 2.07 ab	71.15 ± 1.97 b	71.22 ± 2.04 b	70.88 ± 2.85 b	70.03 ± 1.98 bc	67.34 ± 1.85 c	55.43 ± 2.56 d
SCSH-9-6/12	73.42 ± 1.5 a	70.06 ± 3.03 ab	68.66 ± 2.45 b	62.34 ± 2.2 c	60.26 ± 1.94 cd	59.43 ± 2.66 cde	58.28 ± 1.38 de	56.25 ± 1.8 e
SCSH-9-10/12	44.95 ± 1.55 a	44.25 ± 1.66 ab	44.59 ± 2.48 ab	42.37 ± 2.22 ab	41.99 ± 1.36 b	38.27 ± 1.6 c	23.25 ± 1.85 d	23.22 ± 1.29 d
SCSH-9-11/12	54 ± 2.07 a	53.76 ± 1.69 a	53.18 ± 1.43 a	53.07 ± 1.31 a	52.55 ± 1.84 a	52.19 ± 2 a	46.25 ± 2.02 b	47.28 ± 1.34 b
SCSH-9-17/12	48.22 ± 2.62 a	48.02 ± 2.31 a	45.43 ± 2.25 b	44.22 ± 0.65 b	40.21 ± 3.08 c	40.33 ± 3.03 c	38.23 ± 2.42 d	38.23 ± 2.57 d
SCSH-11-9/13	72.87 ± 1.56 a	69.27 ± 1.3 ab	68.42 ± 3.15 bc	65.25 ± 1.11 c	60.93 ± 1.59 d	58.23 ± 2.07 de	52.1 ± 2.35 e	46.94 ± 1.37 f
SCSH-11-11/12	52.57 ± 1.57 a	46.23 ± 2.69 b	38.21 ± 1.43 c	37.86 ± 1.61 cd	36.23 ± 1.58 cd	36.3 ± 1.45 cd	36.06 ± 2.24 cd	35.45 ± 1.4 d
SCSH-11-15/12	54.38 ± 1.78 ab	54.77 ± 3.1 a	53.32 ± 1.3 abc	52.11 ± 0.73 abcd	50.58 ± 2.13 bcd	50.42 ± 1.05 cd	50.23 ± 0.77 cd	49.03 ± 1.32 d
SCSH-13-4/13	59.83 ± 2.58 a	57.33 ± 1.31 a	57.51 ± 0.97 a	50.66 ± 1.64 b	48.78 ± 0.96 bc	46.32 ± 0.4 c	47.25 ± 1.24 bc	42.22 ± 1.11 d
SCSH-13-17/12	50.77 ± 1.14 a	51.23 ± 1.82 a	49.62 ± 1.49 ab	47.37 ± 1.79 b	47.3 ± 1.53 b	42.87 ± 2.64 c	40.42 ± 1.62 c	40.23 ± 2.1 c
SCSH-15-7/12	55.54 ± 1.67 a	55.43 ± 1.25 a	43.23 ± 1.12 c	47.37 ± 1.33 b	42.23 ± 1.42 cd	42.35 ± 2.1 cd	41.19 ± 1.74 cd	40.4 ± 2.32 d
SCSH-17-8/14	51.25 ± 2.07 a	52.52 ± 1.44 a	48.42 ± 1.61 b	38.92 ± 1.88 c	37.43 ± 1.5 c	37.39 ± 1.87 c	37.33 ± 0.86 c	32.25 ± 1.26 d
SCSH-17-19/13	60.23 ± 1.18 a	62.56 ± 1.22 a	60.21 ± 1.32 a	44.52 ± 0.94 b	37.86 ± 0.62 c	36.43 ± 2.26 c	33.22 ± 0.85 d	33 ± 1.99 d

H—harvesting stage. Results are means of four determinations ± SEm. Different letters within a row followed by Mean ± SEm show a significant difference at *p* ≤ 0.05.

**Table 3:** Relationship between hedonic score, TSS/TA ratio, and BrimA estimated as R^2^ for linear regression line for various harvesting stages in citrus hybrids.

Hybrid Genotypes	R^2^ (Coefficient of Determination)	
	Hedonic Score and TSS/TA Ratio	Hedonic Score and BrimA
SCSH-5-10/12	0.33	0.46
SCSH-7-2/12	0.27	0.31
SCSH-7-7/13	0.39	0.57
SCSH-9-2/12	0.01	0.02
SCSH-9-6/12	0.14	0.32
SCSH-9-10/12	0.18	0.25
SCSH-9-11/12	0.02	0.02
SCSH-9-17/12	0.26	0.41
SCSH-11-9/13	0.72	0.78
SCSH-11-11/12	0.27	0.45
SCSH-11-15/12	0.46	0.48
SCSH-13-4/13	0.52	0.58
SCSH-13-17/12	0.38	0.42
SCSH-15-7/12	0.38	0.46
SCSH-17-8/14	0.76	0.78
SCSH-17-19/13	0.73	0.78

**Table 4:** Description of interspecific citrus scion hybrids evaluated under present study.

Sr. No.	Hybrid Genotypes	Parentage	Year of Planting
1	SCSH-5-10/12	White Pummelo × Mosambi	2012
2	SCSH-7-2/12	White Pummelo × Mosambi	2012
3	SCSH-7-7/13	White Pummelo × Mosambi	2013
4	SCSH-9-2/12	Red Pummelo × Mosambi	2012
5	SCSH-9-6/12	White Pummelo × Mosambi	2012
6	SCSH-9-10/12	White Pummelo × Mosambi	2012
7	SCSH-9-11/12	White Pummelo × Mosambi	2012
8	SCSH-9-17/12	White Pummelo × Mosambi	2012
9	SCSH-11-9/13	White Pummelo × Mosambi	2013
10	SCSH-11-11/12	White Pummelo × Mosambi	2012
11	SCSH-11-15/12	White Pummelo × Mosambi	2012
12	SCSH-13-4/13	White Pummelo × Mosambi	2013
13	SCSH-13-17/12	White Pummelo × Mosambi	2012
14	SCSH-15-7/12	White Pummelo × Mosambi	2012
15	SCSH-17-8/14	White Pummelo × Mosambi	2014
16	SCSH-17-19/13	White Pummelo × Mosambi	2013

## Data Availability

All data supporting the findings of this study are available within the paper.
